# Evaluating the effectiveness of a CRSCE-based de-escalation training program among psychiatric nurses: a study protocol for a cluster randomized controlled trial

**DOI:** 10.1186/s12913-020-05506-w

**Published:** 2020-07-10

**Authors:** Junrong Ye, Aixiang Xiao, Chen Wang, Zhichun Xia, Lin Yu, Sijue Li, Jiankui Lin, Yao Liao, Yu Xu, Yun Lei Zhang

**Affiliations:** 1 Department of Nursing Administration, Affiliated Brain Hospital of Guangzhou Medical University (Guangzhou Huiai Hospital), Guangzhou, 510370 China; 2 Department of Social Psychiatry, Affiliated Brain Hospital of Guangzhou Medical University (Guangzhou Huiai Hospital), Guangzhou, China; 3 Department of Traditional Chinese Medicine, Affiliated Brain Hospital of Guangzhou Medical University (Guangzhou Huiai Hospital), Guangzhou, China; 4 Department of Early Intervention, Affiliated Brain Hospital of Guangzhou Medical University (Guangzhou Huiai Hospital), Guangzhou, China; 5 Department of Adult Psychiatry, Affiliated Brain Hospital of Guangzhou Medical University (Guangzhou Huiai Hospital), Guangzhou, China; 6 Department of Cardiothoracic Surgery, Jingzhou Central Hospital, Jingzhou, China; 7 Department of Intensive Care Unit, West China Hospital of Sichuan University, Chengdu, China

**Keywords:** Cluster randomized controlled trial, De-escalation, Protocol, Training program

## Abstract

**Background:**

The high incidence of workplace violence (WPV) in clinical mental health settings has caused a series of negative impacts on nurses, which has subsequently increased public concern. De-escalation (DE) is recommended as a training program which aims at providing nurses with skills and strategies to more effectively respond and manage WPV. Very few studies have examined the effectiveness of DE training, with current studies possessing various limitations due to their design and small sample sizes. By using a cluster randomized controlled design, the proposed study aims to evaluate the effectiveness of a CRCSE-based DE training programs among psychiatric nurses.

**Method:**

A cluster randomized controlled trial, with a 6-month follow-up period after the end of the intervention, will be conducted among psychiatric hospitals in Guangdong, China. The randomization unit is each involved psychiatric hospital. Participants in the control group will be assigned to routine WPV management training, participants of the intervention group will undergo the same training while additionally receiving DE training. The DE training will include the following five modules: communication, response, solution, care, and environment (CRSCE). Primary outcomes are objective clinical indicators, which will be extracted from the information systems of the enrolled hospitals. These include the incidence of WPV, injuries caused by WPV, and the use of coercion (physical restraint and seclusion) by nurses. Secondary outcomes, aims at evaluating the effects of DE training on nurses, include the capacity of DE, DE confidence, level of job burnout, and professional quality of life. Data will be collected at baseline (T_0_), at 3 months (T_1_, intervention completed), and at 6 months after intervention (T_2_, follow-up).

**Discussion:**

This study will offer trial-based evidence of the efficacy of a DE training program targeted at WPV among psychiatric nurses. DE training is expected to reduce both the total incidence and negative impacts of WPV, with additional improvements in psychiatric nurses’ coping skills.

**Trial registration:**

Chinese Clinical Trial Registry, ChiCTR1900022211. Prospectively registered on 30 March 2019.

## Background

Workplace violence (WPV) includes any act or threat occurring at a person’s work site involving physical violence, harassment, intimidation, and other threatening or disruptive behaviors [1]. WPV towards healthcare workers has become a warning global issue, with the incidence varying from 8 to 38% [2]. WPV can occur in various manners, ranging from verbal threats to physical assaults. Noticeably, WPV against medical workers is greater in psychiatric wards than in other departments (emergency department, medical and surgical wards, outpatient, and laboratory service) [3]. It has been asserted that, among psychiatric nurses, the incidence of WPV during their career was approximately 100% [4, 5]. Other studies reported that approximately 56.1 to 70% of psychiatric nurses expressed experiences of being physically assaulted by patients at the workplace [6, 7]. In China, WPV-related issues have become increasingly serious, with approximately 82.4 to 94.6% of Chinese psychiatric nurses reporting that they had suffered from at least one type of WPV in the last year, with the incidences of verbal abuse, physical assaults, and sexual harassment being 78.6 to 92.1%, 61.5 to 81.9%, and 18.6 to 42.9%, respectively [8, 9].

The high incidence of WPV in mental health settings has caused a series of negative impacts among nurses, with exposure to WPV usually leading to physical injuries. An investigation by Yang et al., [9] noted that perpetrators, who had physically assaulted psychiatric nurses, had done so by bumping, pushing, gripping, biting, and even punching them. A survey among psychiatric nurses showed that 26% of them were seriously injured due to WPV, sustaining wounds like skin injuries, eye injuries, bone fractures, and permanent disabilities [10]. Moreover, a recent study reported that WPV contributed to increases in sick-leave taking among psychiatric nurses [11]. Besides physical injuries, Needham et al. [12] discovered that WPV also caused certain critical, non-somatic negative effects on psychiatric nurses. Exposure to WPV resulted in bio-physiological (depression, anxiety, and fear), cognitive (thinking of oneself as disrespected, violated, threatened, and robbed of one’s rights), emotional (anger, exhaustion, guilt, and self-blame), and social effects (doubts on job appropriateness and sense of insecurity) among psychiatric nurses, with some participants being diagnosed with post-traumatic stress disorder (PTSD). Approximately 75% of assaulted psychiatric nurses had complained that WPV had caused psychological burden [13]. Studies congruously claimed that WPV was a vital factor leading to anxiety (r = 0.242, *P* < 0.01) and depression (r = 0.115, *P* < 0.01) [13–15]. A recent study verified that WPV was significantly correlated with a higher job burnout level among nurses because the exposure to it led to both depersonalization and emotional exhaustion [9]. Another study showed that, among psychiatric nurses, exposure to WPV increased work-based stress and job burnout, and reduced their professional quality of life as a direct consequence [7].

The high incidence and severe impacts of WPV has subsequently increased public concern around it. Empirical findings summarized WPV as being associated with the characteristics of patients, nurses, institutes, and general society [8, 14, 16–20]. On one hand, patients with mental illnesses, poor treatment adherence, substance abuse, and aggressive behaviors were found to have a higher risk of posing WPV against psychiatric nurses [21]. Furthermore, nurses who had less work experience, less training, lacked de-escalating techniques, engaged in rude behaviors, and were mentally unwell [13, 15], were found to more frequently suffer WPV [19]. Additionally, empirical studies have also found that those wards with low quality environments and poor decorations resulted in patients’ unpleasant experiences and views around being hospitalized, which would be expressed via their WPV [22, 23]. Furthermore, socio-cultural factors set the tone for the overall social atmosphere influencing public attitudes on violence, following which, the acquiescence of violent behaviors from local cultures may have exacerbated WPV incidences against nurses [19].

Having discovered various factors contributing to WPV, strategies reducing its incidence and negative impacts have been developed accordingly. Occupational Safety and Health Administration (OSHA) guideline indicates that WPV prevention program should be based on the simultaneous implementation of organizational measures, administrative measures, and staff training [24]. Interventions targeted at enhancing staffing levels, restructuring the workplace environments, promoting work organization, improving nurse-patient therapeutic relations, and training medical workers have all proven effective in alleviating WPV [19, 20, 25, 26]. Notably, substantial evidence has found that the promotion of improved de-escalation skills among psychiatric nurses is the most widely acknowledged intervention strategy [22, 27–33]. Experts have reached a consensus that de-escalation (DE) technique is the first-line WPV management strategy during early incidence stages [34, 35]. DE refers to a range of skills involving averting conflicts through emotional regulation and self-management techniques [36]. In the psychiatric nursing context, studies have shed light on the contents of DE. It has been proposed that DE should focus on nurse-patient interactions, patient engagements, choosing appropriate intervention times, ensuring mutual safety, confirming patients’ autonomy, limit-setting, and authoritative interventions [37, 38]. Another study by Hallett and Dickens [39], using concept analysis, emphasized that DE was achieved through techniques around self-regulation, communication, assessment, action, and safety maintenance [39]. The application of DE has had positive impacts on improving nurses’ capacities, as well as on nurse-patient relationships, while reducing the use of coercive strategies by nurses (including seclusion, physical restraints, and mechanical restraints). After comparing pre- and post- results of the De-escalating Aggressive Behavior Scale, Nau et al., [40] suggested that the training of DE enhanced student nurses’ performances in dealing with WPV (with scores for untrained = 3.01 vs. trained = 3.61). Another study also found that nurses receiving DE training possessed greater self-perceived confidence than those without, demonstrating that this form of preparation effectively enhanced nurses’ performances by strengthening their confidence levels in dealing with WPV [41]. In addition, Fröhlich et al., [42] highlighted DE training’s role in helping to cultivate a harmonious atmosphere within psychiatric wards by improving patients’ coherence and subjective safety. Most importantly, the application of DE techniques decreases the use of coercive strategies. Empirical studies verified that DE training, targeting psychiatric nurses, reduced the use of physical restraints by approximately 26.4 to 74.0%, and had cut down their likely duration and resultant adverse effects [43]. Current findings have confirmed that DE is a significantly utilized approach in dealing with WPV as well as in reducing the use of coercion, suggesting that DE is an indispensable component of standardized training programs for psychiatric nurses [29, 41, 44, 45]. However, the presented studies all possesses some limitations. First, among most studies, DE training is combined with other components concurrently, such as improved staffing levels, leadership styles, and risk assessments, and, therefore, the actual efficacy of DE itself remains unclear. Second, studies concerning DE provides weak creditability due to their lack of trial-based evidence [46]. Third, the characteristics of the DE training programs used among the current studies varies from one to another because its design needs to be matched to the environmental and socio-cultural factors— hence, the findings of Western studies might not be accurate in China. Last, very few studies simultaneously appraise the effectiveness of DE training via the improvement of nurses’ capacities and objective indicators.

### Objectives

Based on a literature review, and through using the Delphi method, a program has been developed in order to improve nurses’ WPV coping capacities. This program is named after the first letter of each core component, which are Communication, Response, Solution, Care, and Environment (CRSCE), respectively. The aim of this study is to evaluate the effectiveness of a DE training program using the CRSCE core components among Chinese psychiatric nurses.

## Methods

### Trial design

The CRSCE program is a multi-center, single blinded, cluster randomized controlled trial with a 6-month follow-up period. All stages of this trial are in accordance with the CONSORT guidelines.

### Settings and participants

This trial will be conducted among 6 major public psychiatric hospitals in Guangdong, China. The involved psychiatric hospitals are Guangzhou Mental Health Center (GZ), Shenzhen Mental Health Center (SZ), the Third Hospital of Foshan City (FS), the Second Hospital of Huizhou (HZ), Shantou Mental Health Center (ST), and the Third Hospital of Meizhou (MZ). The number of wards (secured and non-secured) and nurses of the involved hospitals are shown in Table 1*.* This study has gained ethical approval from the IRB of GZ. Executives from the involved institutes will be informed and asked for their permission to conduct the study, with the help of the nurses. Informed consent will be obtained from all participants before they complete the surveys.

**Table 1:** Numbers of wards and nurses of recruited hospitals (secured and non-secured)

Hospital	Non-secured ward			Secured ward		
	Number of wards	Number of beds	Number of nurses	Number of wards	Number of beds	Number of nurses
GZ	3	120	65	14	865	322
SZ	1	45	18	10	623	297
FS	1	106	29	8	816	243
HZ	1	44	20	7	906	291
ST	3	60	46	8	620	231
MZ	1	60	20	7	700	220

### Inclusion criteria

Aged from 18 to 60 years.

Registered nurses engaging in mental healthcare.

Are employed as full-time nurses.

### Exclusion criteria

Student nurses and nursing interns.

Personnel taking refresher trainings at engaged wards.

### Interventions

Participants in the control group will receive routine WPV management training, participants of the intervention group will undergo the same training while additionally receiving CRSCE-based training. CRSCE is a 5-module training program, composing 104 learning hours, which will be completed in 3 months. The modules, objectives, and learning hours of routine WPV management training and the CRSCE program are presented in Table 2.

**Table 2:** Modules, objectives, and learning hours of routine WPV management training and CRSCE

Module		Content	Objects	Hours		Routine WPV management training	CRSCE training
				Lecture	Practice		
Routine WPV management training		Basic Communication Skills of Nursing	To understand the concept of communication skills and its attributes To understand different types of communication skills To learn how to interact with patients in practice	4	–	**✓**	**✓**
		Communication Skills in Mental Health Care	To identify the attribute of communication skills in mental health care To distinguish the difference of communication skills between mental health and general nursing To learn how to interact with psychiatric patients	4	4	**✓**	**✓**
		Risk Assessment of Violence	To understand the types of WPV To learn how to use different assessment tools To discuss advantage and disadvantage of assessment tools	4	–	**✓**	**✓**
		The Ethic and Law in Metal Health Care	To discuss the ethical issues in mental health care To discuss how nurses to balance the ethical issues and law in mental health care	4	–	**✓**	**✓**
		De-escalation	To know the concept of De-escalation To identify the attributes of De-escalation To discuss the key components contribute to successful de-escalation	4	–	**✓**	**✓**
		Practical WPV coping skills	To learn the breakaway techniques, holding methods To learn the control and restraint methods	4	8	**✓**	**✓**
CRSCE training	Communication	How to Build the Therapeutic Nurse-Patient Relationship	To identify the factors that influence therapeutic relationship To identify the key components of building the therapeutic relationship with patients To learn how to build therapeutic nurse-patient relationship using communication skills	4	4	**NA**	**✓**
		The Communication Skills with Aggressive Patients	To learn the communication skills with aggressive patients	4	4	**NA**	**✓**
	Response	The Early Stage Signal of WPV	To identify the early stage signal of WPV To discuss and learn what a nurse should do when he/she has identified a patient is in WPV early stage	4	4	**NA**	**✓**
		What Is Your FIRST Reaction When WPV Happens?	To recall nurses’ memories of facing violence To share nurses’ experience of coping with violence To refresh and discuss the appropriate method to manage WPV	4	–	**NA**	**✓**
		When WPV Happens, What Should We Do?	To learn how to response WPV To discuss the alternatives of WPV	4	2	**NA**	**✓**
		What Is the Influence of WPV on You?	To discuss and share the influence of WPV on individual	4	–	**NA**	**✓**
	Solution-Focused Technique	Cognitive Positive Psychology	To learn the concept of cognitive positive psychology and how to use it in clinical work	4	4	**NA**	**✓**
		The Concept and Principle of Solution-Focused Technique in Nursing	To learn the concept and principle of solution-focused technique	4	–	**NA**	**✓**
		The Five Stages of Psychological Intervention of Solution-Focused Technique and Its Application	To identify the five stages of psychological intervention of solution-focused technique To learn using the five stages of psychological intervention in practice	4	2	**NA**	**✓**
	Care	The History and Development of Humane Care Service	To understand the history and development of humane care service To identify the key elements of humane care service	4	–	**NA**	**✓**
		The Relationship Between Humane Care Service and WPV	To discuss the relationship between humane care service and WPV To learn proving humane care to aggressive patients	4	4	**NA**	**✓**
		The Humane Care Service in Mental Health Care	To identify the humane care service in mental health care To discuss what nurses can do to provide the humane care service to psychiatric patients	4	4	**NA**	**✓**
	Environment	The Innovation of Environment in Mental Health Care	To understand the concept of environment in mental health care To understand the change process and development of environment in mental health care	8	4	**NA**	**✓**
		Evidence Base Practice: The Relationship Between Ward Environment and WPV	To discuss how environment affects the WPV To find out the environmental hazards of WPV through literature review To implement achievable improvement of ward environment	8	8	**NA**	**✓**
**✓**: Included modules **NA**: Not Available				Total Learning Hours		**36 h**	**140 h**

### Outcomes

Primary outcomes are objective clinical indicators of the included wards, which will be extracted from the hospital information systems and their annual reports. The objective indicators include the frequency of WPV, injuries caused by WPV, and the use of coercion (physical restraint and seclusion). The objective indicators will be calculated as follows:

- monthly WPV frequency = monthly numbers of WPV event / total monthly patient days × 1000 ‰;
- monthly frequency of injuries caused by WPV = monthly numbers of injuries caused by WPV / total monthly patient days × 1000 ‰ and;
- monthly frequency of physical restraint (or seclusion) = monthly numbers of patient days of physical restraint (or seclusion) / total monthly patient days × 1000 ‰.

**Fig. 1:**
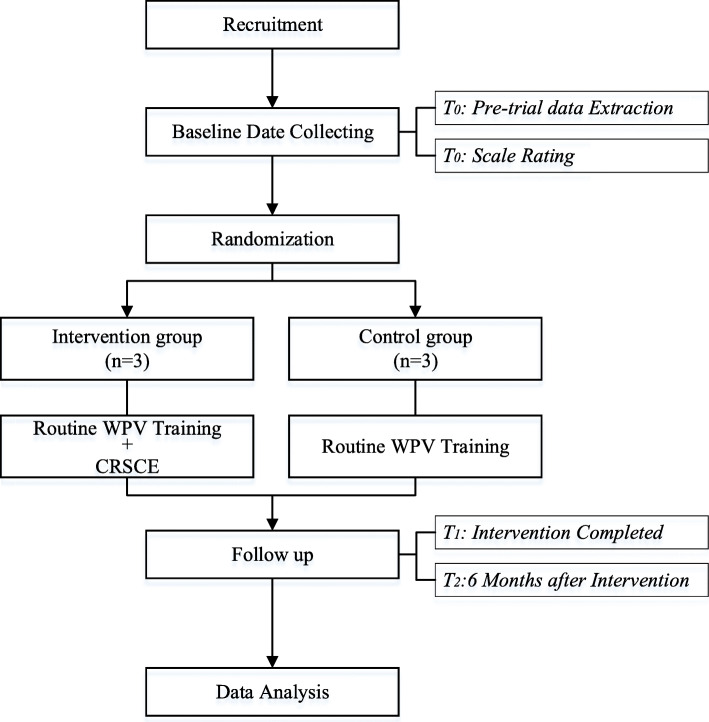
Trial Flow Chart

Secondary outcomes are collected in order to evaluate the impacts on the nurses. The De-escalating Aggressive Behavior Scale (DABS), Confidence in Coping with Patient Aggression Instrument (CCPAI), Maslach Burnout Inventory-General Survey (MBI-GS), and Professional Quality of Life Scale (Pro QOL), will all be used to evaluate the capacity of DE, confidence of DE, level of job burnout, and professional quality of life, respectively. The above survey instruments will be used after obtaining licenses. The flow chart of this study is presented in Fig. 1.

### Sample size

Sample size estimation is based on monthly frequency of WPV. Using the study by I. et al., (2004), we set a significant change of 12.1% (decreased from 17.8% by 5.70%), alpha error of 0.05, beta error of 0.20, the estimated number (N _simple_) of monthly records is 29 per group for simple randomized control trial [47]. The intra-class correlation coefficients (ICC), explains the inflation factor of cluster randomized controlled design trial, is set as 0.05 in proposed study [48]. Based on the numbers of wards of MZ and HZ (*n* = 8), the minimum number of 6-month follow-up record (m) is 48. The sampling size of cluster randomized controlled trial (N _cluster_) is calculated as: N _cluster_ = [1 + (m^− 1^) * ICC] * N _simple_ ≈ 98. The minimum number of recruited hospitals of each group (N) is calculated as: N = N _cluster_/ m ≈ 2.04 < 3, suggesting at least 3 hospitals per group.

### Randomization

This is a cluster randomized controlled trial. The randomization unit is every involved psychiatric hospital. Involved psychiatric hospitals will be consecutively coded from 1 to 6 by a statistician not actively engaging in this study. By using an online random number generator, hospitals will be assigned to either the intervention or control group according to a 1:1 ratio. The statistician will then inform the research coordinator of the group allocations. Afterwards, the training schedule of each hospital will be designed according to its allocation. To ensure justice, when the intervention group has completed the CRSCE-based program, and follow-up data have been collected, the control group will also receive the same CRSCE-based training.

### Blinding

This is a single-blinded study. Engaged nurses and their managers will not be aware of their hospital’s allocation. The surveys will be completed by research assistants who are not involved in the CRSCE training program.

### Data collection

Primary outcomes will be continuously collected every month by extracting data from the hospital information systems (HIS) and their annual reports. Secondary outcomes are collected at baseline (T_0_), 3 months after intervention (T_1_, the end of intervention), and 6 months after intervention (T_2_, follow-up). Data collection will be completed by research assistants who are not aware of this study’s design.

### Statistical methods

Statistical analysis will be performed using SPSS version 22.0 software (SPSS Inc., Chicago, IL, USA). Descriptive statistics will be reported as frequencies and percentages, if applicable. The Shapiro-Wilk test will be used in order to examine the distributions of the continuous outcomes. Quantitative variables will be presented using means and standard deviations or as the median and interquartile range. A Student’s t-test, Mann-Whitney U test, Chi-square test, or Fisher’s exact test will be adopted to compare the groups according to their normality distributions. Additionally, a repeated ANOVA will be used to explore the effectiveness of the CRSCE training program and further regression analysis will be performed, if appropriate. The statistical significance will be set at *P* < 0.05, two tailed, with a 95% confidence interval (*CI*).

### Study quality control

All instruments used in this study have been examined for their validity and reliability. Part-time nurses are not available among the 6 involved institutes. Involved psychiatric hospitals are located in different cities of Guangdong Province, and therefore possible contamination between groups is unlikely. To improve the homogeneity of the intervention, during this study newly employed nurses of the involved institutes will be assigned to the training program and will be evaluated accordingly. Short-duration refresher courses will be monthly arranged to maintain accreditation and competency among psychiatric nurses of intervention group.

## Discussion

WPV is prevalent in psychiatric hospitals, resulting in critically adverse impacts on nurses. This study will examine the effectiveness of interventions of reducing these WPV impacts. DE training is a recommended intervention for helping psychiatric nurses in dealing with WPV, but more trial-based evidences are needed in order to support its effectiveness. To the best of our knowledge, this is the first study protocol evaluating the effectiveness of DE using a cluster randomized controlled trial. Compared to routine WPV management training, CRSCE is an additional and innovative training program for psychiatric nurses. The modules of CRSCE are expected to address the crucial components of DE, as well as the general mental health service backgrounds in China. Usually, the patient’s unmet demands are found to be a prominent cause of WPV; therefore, solution-oriented and humane care approaches are warranted, with these being achieved by appropriate communications and responses by healthcare professionals. In addition, therapeutic environments also have been found to influence the occurrence of WPV. Poor therapeutic environments have been found to cause greater numbers of complaints by patients around hospitals and medical staff, which, to some extent, could result in greater WPV incidences [24]. In China, the governmental investment into mental health services was limited [49], with this inadequate financial support hindering the upgrading of therapeutic environments in psychiatric hospitals. Thus, this is why a therapeutic environment module is to be included in this study. The major strength of this study is that all of the included modules (Communication, Response, Solution, Care, and Environment) have been examined by empirical researches. Study by Magnavita [26] highlighted that effective communication, WPV preventing solution, response by identifying violence-prone individuals, and rearranging environment, were crucial components reducing WPV (assault rate declined from 0.24 ± 0.02 to 0.04 ± 0.03, *t* = 14.4, *P* < 0.01) in psychiatric units [26]. In addition to the components mentioned by Magnavita [26], OSHA WPV preventing guideline and Irish physical reduction strategy simultaneously propose, providing humane care (i.e. trauma informed care) is a successfully instituted approach in psychiatric hospitals in preventing WPV and reducing the use of physical restraint [24, 50]. Therefore, the employed modules are expected to reduce WPV incidence and WPV resultant impacts.

The outcomes of the CRSCE training program, as analyzed by this study, will present helpful and practical evidence for healthcare providers and policy makers. The primary outcome data used will be the objective indicators of WPV, extracted from hospital incident data, and this will contribute to the evaluation of the CRSCE training program. The secondary outcome will assess the influence of CRSCE training program on nurses by using questionnaires. These results will inform practitioners and policy makers as to how and to what extent CRSCE improves psychiatric nurses’ WPV coping capacities. In proposed study, we hypothesize the frequency of WPV, of injuries caused by WPV, and of coercion (physical restraint and seclusion), will be decreased because CRSCE training program would help preventing WPV in early stage. Besides, after intervention, we are expected to see the increased scores of DABS, CCPAI, and Pro QOL; and a significant decline in MBI-GS score. These might reflect CRSCE training program improves the capacity and confidence in dealing with WPV, and helps to relieve job burnout symptom.

However, this study has a few limitations. First, this study only includes psychiatric nurses. Psychiatrists, unlicensed nursing assistants (UNA), and other professionals will not be recruited. However, the practices of UNA are supervised by registered nurses and WPV alleviation is usually directed by nurses, meaning that psychiatric nurses are the most affected population group in this regard. Thus, training involving registered psychiatric nurses will be the most apropos approach. Second, this study applies a single-blinded design, and, as a result, the CRSCE instructors might be aware of their own group allocation because they will be knowledgeable of the structure of both the CRSCE and routine training programs. To eliminate this bias, primary outcome recordings and surveys will be completed by research assistants who are not aware of this study’s design. Third, CRSCE training program might affect the reporting behavior because it might enhance WPV awareness among psychiatric nurses, which would influence the generalization of research data in result. This reporting bias would be controlled to the most extent as all recruited hospitals are required to follow the national guideline of reporting nursing indicators [51]. Last, uncontrollable factors contributing to increased rates of aggression might influence the objective indicators. Despite the limitations above, this study is expected to evaluate the effectiveness of the CRSCE training program on WPV alleviation and its inherent benefits for nurses.

### Trial status

The proposed trial had been prospectively registered at the Chinese Clinical Trial Registry (Registration Number: ChiCTR1900022211).

## Data Availability

The datasets analyzed during the current study are not publicly available due because further data analysis may be undergoing, but are available from the corresponding author on reasonable request.

## Abbreviations

CCPAI: Confidence in Coping with Patient Aggression Instrument
DABS: De-escalating Aggressive Behavior Scale
DE: De-escalation
MBI-GS: Maslach Burnout Inventory-General Survey
Pro QOL: Professional Quality of Life Scale
WPV: Workplace violence
